# Rescue Vaginal Cerclage to Stop Funneling Following Laparoscopic Cerclage

**DOI:** 10.1055/s-0041-1736553

**Published:** 2021-11-16

**Authors:** Tayfun Cok

**Affiliations:** 1 Department of Obstetrics and Gynecology, School of Medicine, Baskent University, Adana, Turkey

Dear Editor,

Laparoscopic cerclage is an effective treatment option for cervical insufficiency leading to repeated preterm birth. However, surgical intervention with various cerclage techniques, such as vaginal, transabdominal laparoscopic approaches, still remains the ultimate solution, unfortunately, without the guarantee of success. 1 2 3 There is still no consensus regarding the priority of each technique over the other. However, when laparoscopic cerclage fails to completely treat cervical insufficiency, an additional vaginal cerclage should be considered as a rescue intervention. We suggest considering Shirodkar vaginal cerclage a rescue technique following laparoscopic transabdominal cerclage which is compromised by further funneling. Here, we report, after obtaining written consent, the cases of three patients who needed additional vaginal cerclage to prevent further funneling and membranous bulging despite intact laparoscopic cerclage material.

These three patients had recurrent pregnancy loss despite having undergone vaginal cerclages. Demographic data, as well and the obstetric and surgical histories of the patients, are shown in Table 1 . Considering their history, the first preferred intervention was laparoscopic cerclage. However, we detected funneling and bulging of amniotic membranes below the level of the laparoscopic cerclage during their follow-up visits. Then, we performed an additional Shirodkar vaginal cerclage to prevent further funneling. The images of the patients' cervix immediately after the Shirodkar cerclage are shown in Fig. 1 . The patients were followed-up with frequent ultrasound (US) examinations; images of funneling following vaginal cerclage persisted in two patients, whereas funneling disappeared completely in one patient after vaginal cerclage. All patients had uneventful deliveries at 38 weeks.

**Table 1:** Demographic data, and obstetric and surgical histories of the patients

Patient	1	2	3
Age	36	33	34
Gravida	10	5	3
Para	1	1	0
Abortus	8	3	2
Previous gynecological operation	Septum resection	None	None
Live birth	1 at 28 weeks	1 at 30 weeks	None
Number of previous elective McDonald vaginal cerclages	3	2	1
L/S cerclage	+	+	+
Issue	Funneling	Funneling	Funneling
Week at performance of vaginal Shirodhar cerclage	13 weeks, 5 days	23 weeks, 2 days	26 weeks, 1 day
Delivery at	38 weeks, 3 days	38 weeks, 1 day	38 weeks, 2 days

**Fig. 1:**
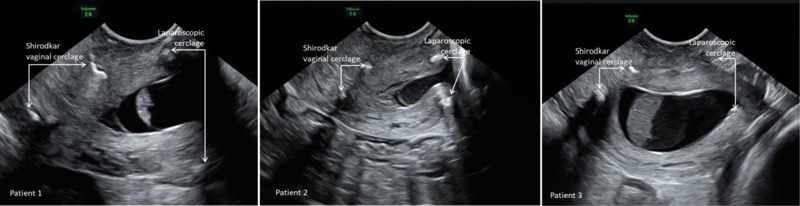
Ultrasonography images of the patients' cervixes after Shirodkar vaginal cerclage.

Laparoscopic abdominal cerclage is an effective management option for refractory cervical insufficiency. It is reported to improve the rates of second-trimester loss and neonatal survival, 4 and to be superior to low vaginal cerclage, especially for patients with failed previous vaginal cerclage. 5 However, it can be insufficient in conditions such as laparoscopic interventions with loose first knots or medial deviation into the cervical stroma during suturation, or vaginal infections. Further funneling and bulging of amniotic membranes can be warning signs of pregnancy loss even after an uneventful and intact laparoscopic cerclage. This condition can be due to congenital or acquired cervical tissue defects, previous repeated surgeries of the cervix, or a lax laparoscopic cerclage. In these cases, we preferred to supplement the previous laparoscopic cerclage with a subsequent vaginal one through the Shirodkar technique, which is performed at a higher level of the cervix compared with the McDonald technique. This intervention refortified the cervix mechanically for further dilatation. We suggest that the alternative use of this well-known technique may be considered in such difficult cases to provide live births for patients with long history of pregnancy loss.

**Reply to Letter to the Editor**

## Comments by the President of the National Commission Specialized in High-Risk Pregnancy (Febrasgo)

Rosiane Mattar ^1^ 0000-0003-1405-5371

^1^ Escola Paulista de Medicina, Universidade Federal de São Paulo, São Paulo, SP, Brazil

**Address for correspondence** Rosiane Mattar, MD, Rua Botucatu, 740, Vila Clementino, São Paulo, SP, 04023-062, Brazil (e-mail: rosiane.toco@epm.br).

Abdominal cerclage should be restricted to cases in which it is impossible to perform the procedure vaginally, as it leads to greater maternal morbidity: it determines a greater risk of bleeding, infection, rupture of the membranes, and cesarean section. I think that, if Shirodkar cerclage was possible after laparoscopic surgery, it should have been the first treatment option, which would reduce the risks and guarantee success. In addition, the fact that the funnel appeared after surgery shows that the tape was not properly tightened in the suture via the abdominal route, keeping the canal widened, as if the cerclage had not been performed. Thus, cerclage via the abdominal route should be very well indicated and very well performed when necessary.

**Conflicts of Interests**

The author has no conflict of interests to declare.
